# Post-implantation clinical cost analysis between transcutaneous and percutaneous bone conduction devices

**DOI:** 10.1007/s00405-023-08099-2

**Published:** 2023-07-08

**Authors:** Tjerk W. Aukema, Emma M. Teunissen, Arno M. Janssen, Myrthe K. S. Hol, Emmanuel A. M. Mylanus

**Affiliations:** 1 Department of Otorhinolaryngology/Head and Neck Surgery, University Medical Center Groningen, PO Box 30.001, 9700 RB Groningen, The Netherlands; 2 Research School of Behavioral and Cognitive Neurosciences, Graduate School of Medical Sciences, University of Groningen, Groningen, The Netherlands; 3 Department of Otorhinolaryngology/Head and Neck Surgery, Radboud University Medical Center, Nijmegen, The Netherlands; 4 Donders Center for Neuroscience, Radboud University, Nijmegen, The Netherlands

**Keywords:** Hearing loss, Bone conduction device, Transcutaneous, Percutaneous, Hearing aids

## Abstract

**Introduction:**

Bone conduction devices (BCD) are effective for hearing rehabilitation in patients with conductive and mixed hearing loss or single-sided deafness. Transcutaneous bone conduction devices (tBCD) seem to lead to fewer soft tissue complications than percutaneous BCDs (pBCD) but have other drawbacks such as MRI incompatibility and higher costs. Previous cost analyses have shown a cost advantage of tBCDs. The purpose of this study is to compare long-term post-implantations costs between percutaneous and transcutaneous BCDs.

**Materials and methods:**

Retrospective data from 77 patients implanted in a tertiary referral centre with a pBCD (*n* = 34), tBCD (*n* = 43; passive (t_pas_BCD; *n* = 34) and active (t_act_BCD; *n *= 9) and a reference group who underwent cochlear implantation (CI; *n* = 34), were included in a clinical cost analysis. Post-implantation costs were determined as the sum of consultation (medical and audiological) and additional (all post-operative care) costs. Median (cumulative) costs per device incurred for the different cohorts were compared at 1, 3 and 5 years after implantation.

**Results:**

After 5 years, the total post-implantation costs of the pBCD vs t_pas_BCD were not significantly different (€1550.7 [IQR 1174.6–2797.4] vs €2266.9 [IQR 1314.1–3535.3], *p* = 0.185), nor was there a significant difference between pBCD vs t_act_BCD (€1550.7 [1174.6–2797.4] vs €1428.8 [1277.3–1760.4], *p* = 0.550). Additional post-implantation costs were significantly highest in the t_pas_BCD cohort at all moments of follow-up.

**Conclusion:**

Total costs related to post-operative rehabilitation and treatments are comparable between percutaneous and transcutaneous BCDs up to 5 years after implantation. Complications related to passive transcutaneous bone conduction devices appeared significantly more expensive after implantation due to more frequent explantations.

**Supplementary Information:**

The online version contains supplementary material available at 10.1007/s00405-023-08099-2.

## Introduction

Bone conduction devices (BCD) have proven to be an effective solution for patients with conductive- and mixed hearing loss (CHL; MHL), as well as cases of single-sided deafness (SSD) [1–4]. A percutaneous BCD (pBCD) consists of three parts: (1) a sound processor that can be coupled to (2) a skin-penetrating titanium abutment attached to (3) a titanium implant that is positioned and osseointegrated in the temporal bone.

The most observed complications with a pBCD are primarily soft tissue or skin related (e.g. inflammation and skin overgrowth). Developments in the surgical technique (i.e. subcutaneous tissue preservation), wider implants and longer abutments have led to a decrease in the complication rate [5, 6]. Complications concerning the soft tissue frequently call for a local or topical treatment. In more severe cases, surgical intervention may be necessary or implant loss is observed. Additionally, some patients find pBCDs aesthetically less appealing.

Transcutaneous devices possess the main advantage that the implant is positioned underneath ‘closed skin’, leaving no port d’entrée for dirt and micro-organisms, and are thus less prone to complications [7, 8]. The first transcutaneous BCD (tBCD), the Xomed Audiant, was deemed unsuccessful due to limited maximum sound output and high skin pressure, with concomitant skin-related complications [9, 10]. In the following years, other transcutaneous devices have been developed which may be divided into active and passive types. In passive tBCDs (t_pas_BCD), for instance, the Baha^®^ Attract (Cochlear ltd. Sydney, Australia) and Sophono^®^ (Medtronic, Dublin, Ireland), the sound processor and transducer are attached to the skin using a magnet. Vibrations must pass through the soft tissue to a magnet attached to an implant osseointegrated to the temporal bone. In the available active tBCDs [t_act_BCD; i.e. Bonebridge™ (MED-EL, Innsbruck, Austria); Osia^®^ (Cochlear ltd., Sydney, Australia)], the sound processor is placed outside the skin and the transducer is implanted in the subperiosteal layer, in direct contact with the temporal bone. Sound received by the sound processor is converted and relayed to the internal receiver stimulator using an electromagnetic carrier wave comparable to the technique used in cochlear implants (CIs). Transcutaneous devices have drawbacks such as conditional Magnetic Resonance Imaging (MRI), longer surgical time compared to pBCD and skin pressure due to magnet retention forces.

Amin et al. [11] and Godbehere et al. [12] have investigated the costs of percutaneous and transcutaneous systems and concluded that the initial purchase of a tBCD is more costly, however, due to fewer complications post-implantation—resulting in less treatment—overall costs were lower. In other words: tBCDs seem to become cost-beneficial over time. However, both studies either have a small study population or a relatively short follow-up time. This study compared the total post-implantation costs between pBCDs and tBCDs over 5 years in relatively large groups of patients (*n* = 34).

## Methods

### Study population

Data were collected retrospectively. Patients who underwent t_pas_BCD implantation at our tertiary university medical centre (Radboudumc, Nijmegen, The Netherlands) and met inclusion criteria (adults and completed 5-year follow-up) were identified and included on consecutive basis. This resulted in a cohort of 34 patients implanted between November 2013 and May 2016. Thirty-four adult pBCD patients, consecutively implanted during the same period with a pBCD were selected from an existing database as the control cohort. Nine available adult patients who underwent t_act_BCD implantation and completed 5 years of follow-up were identified and included as well for comparison. t_pas_BCD and t_act_BCD together were referred to as the aggregated tBCD cohort (*n* = 43) and used for analysis. Sub-analysis were performed with the t_pas_BCD and t_act_BCD cohorts separately. As the t_act_BCD has a comparable coupling between external processor and internal transducer as a cochlear implant, a reference cohort of 34 adult consecutive cochlear implant recipients implanted in the same period, was included for sub-analysis.

### Implants and study design

All pBCD patients were implanted with the BI300® osseointegration fixture and BA300® abutment. t_pas_BCD patients were implanted with the BIM400® magnet which was fixed to the cortex of the temporal bone using a BI300® fixture (Baha® Attract). The t_act_BCD cohort received the Osia® 1 system, which is a piezo-electric transducer fixed to the temporal bone with a BI300® fixture. The internal part of the Osia® 1 system consists of two components; the piezo-electric transducer and the implant receiver which is similar to the CI24 platform used for cochlear implants. CI patients were implanted with the Nucleus® (CI422 or CI24RE) system. Cochlear Ltd., Sydney, Australia, manufactured all hearing systems.

Baseline characteristics and demographic data were obtained from medical records. These included gender, age and comorbidities (e.g. diabetes mellitus, intellectual disability, long-term corticosteroid usage, osteoporosis, radiotherapy at the skull, skin diseases).

The total post-implantation costs per cohort were calculated from two sub-categories, namely consultation and additional costs. Firstly, all postoperative consultations with a physician, audiologist or nurse (by telephone and physical) were inventoried. Consults with an audiologist were distanced in a ‘simple’ consultation (e.g. adjustments or replacements of a device) and an ‘extended’ consultation (e.g. speech audiometry, free field testing, etc.). Secondly, all additional costs were calculated and included. These exist out of procedures (e.g. surgeries, revisions, abutment changes, etc.), emergency room (ER) consultations, hospital admissions, and other treatments (e.g. prescribed postoperative care, antibiotics, pain killers, etc.). For the transcutaneous devices, external magnets were included.

Excluded were repairs, since these fall under the warranty of the manufacturer, and personally chosen accessories. At our clinic, after approximately 5 years patients are provided with the opportunity to upgrade their sound processors, but since these are local agreements and processors are not always upgraded at or before our 5-year cut-off, it was decided to exclude these from analysis. Moreover, visits made for research purposes (related to previously performed studies), either medical or audiological, were excluded as well as implant surgery and implant purchase since interest was solely in comparing post-implantation clinical differences. In this study’s medical centre, the default audiological and medical post-implantation rehabilitation protocol of the CI-recipients is different compared to that of the BCDs. Due to the transcutaneous connectivity and tolerance of the CI, post-implantation additional costs were compared.

Costs were compared at 1 (Y1), 3 (Y3) and 5 years (Y5) after implantation, to track differences over time. Explanted patients were not removed from follow-up and the costs made related to the implant until the endpoint (5 years) were included.

### Costs

The costs of consultations and procedures within Dutch hospitals are based on agreements between individual medical centres and the insurance companies they liaise with and base their yearly contracts on, which means they may vary per hospital. The medication prices in this study were obtained from this medical centre’s pharmacy and system prices from the manufacturer’s catalogues (year 2021) (Table 1).

**Table 1:** (a) Overview of costs, (b) prices for implant components used for additional costs

(a)		
Object	Context	Costs (€)
Telephonic consult^a^		
Nurse	Consultation	3.0
Physician	Consultation	65.0
Audiologist	Consultation	65.0
Physical consult^a^		
Nurse	Consultation	0.2
Physician	Consultation	136.0
Audiologist	Consultation	153.0
	Simple fitting and adjustments	222.0
	Extended audiological tests	327.0
Anaesthetist	Consultation	241.0
Local intervention^a^	Abutment change (excl. abutment)	397.0
	Skin revision	269.0
	Local anaesthesia	229.0
Emergency room consult^a^	Seen by a physician at ER	484.0
Ward admission^a^	Overnight stay at hospital	851.0
Surgery (revision)^a^	Operating room (1 h incl. all staff and materials; anaesthesia not defined)	2220.9
Healing cap^c^	1 Cap	43.0
Terra-Cortril + Polymyxin B^b^ (TCPB)	1 Tube ointment	3.0
Fucidin^b^	1 Tube ointment	3.0
Triamcinolone acetonide^b^	1 Tube ointment	2.0
Antibiotics^b^		
Amoxicillin/clavulanic acid	Per tablet (625 mg)	0.1
	Per injection (i.v.; 1200 mg)	1.0
Amoxicillin	Per tablet (500 mg)	0.1
Clindamycin	Per tablet (300 mg)	0.3
Clarithromycin	Per tablet (500 mg)	0.2
Articaine/adrenaline (Ultracain D-S)^b^	Flasc injection fluid	
Oxycodon^b^	Per tablet (5 mg)	0.1
Tramadol^b^	Per tablet (50 mg)	0.02
(b)		

(b)		
Implants and abutments^c^	Context	Costs (€)
pBCD implant	3/4 mm	421.0
pBCD abutment	6/9/12 mm	809.0
t_pas_BCD internal magnet		
External magnet^c^	t_pas_BCD	108.0
	t_act_BCD	32.0
	CI	38.0

All costs are shown rounded

^a^Department of ENT, Radboud University Medical Centre, Nijmegen, The Netherlands

^b^Medical Centre’s Pharmacy, Radboud University Medical Centre, Nijmegen, The Netherlands

^c^Manufactures 2021 catalogue price [excluding Value Added Tax (VAT)], Cochlear ltd., Sydney, Australia

### Statistical analysis

Depending on normality, mean (± SD) or median [IQR] are presented. Unpaired two-tailed t-test or Mann–Whitney U test was performed to assess the statistical significance of differences between device groups at each particular time point (Y1, Y3 and Y5). Between-group differences in baseline characteristics were calculated with one-way ANOVA test. Spearman’s rho was performed to calculate correlations between variables. A *p*-value of 0.05 was considered significant. Data were processed with IBM® SPSS® Statistics version 28.0 (Chicago, IL, USA). Figures were created using GraphPad Prism version 9 (GraphPad Software, Boston, USA).

## Results

### Participants

The study population (37 females and 40 males) consisted of 77 patients with a mean age at implantation of 50.2 years (SD ± 13.8). Mean age at implantation for the pBCDs was 51.6 years (SD ± 15.9), 47.7 years (SD ± 12.5) for the t_pas_BCDs, 54.9 years (SD ± 8.3) for the t_act_BCDs and 55.6 years (SD ± 19.9) for the CI cohort. All patients were implanted unilaterally. Diabetes Mellitus type II (DM II) occurred most frequently (5.4%). Baseline characteristics per cohort are displayed in Table 2. Mental disabilities were significantly more prevalent in the pBCD users (*p* = 0.007).

**Table 2:** Baseline characteristics

Groups	pBCD	t_pas_BCD	t_act_BCD	CI	Total	*p-*value
Total patients	34 (30.9)	34 (30.9)	9 (8.2)	34 (30.9)	111 (100)	
Gender						
Female	16 (47.1)	18 (52.9)	3 (66.7)	22 (64.7)	59 (53.2)	0.296
Male	18 (52.9)	16 (47.1)	6 (33.3)	12 (35.3)	52 (46.8)	
Comorbidities						
DM II	2 (5.9)	1 (2.9)	1 (11.1)	2 (5.9)	6 (5.4)	0.806
Skin disease	2 (5.9)	1 (2.9)	0 (0.0)	1 (2.9)	4 (3.6)	0.525
Radiotherapy	1 (2.9)	1 (2.9)^a^	0 (0.0)	0 (0.0)	2 (1.8)	0.740
Osteoporosis	0 (0.0)	0 (0.0)	0 (0.0)	1 (2.9)	1 (0.9)	
Mental disability	5 (14.7)	0 (0.0)	0 (0.0)	0 (0.0)	5 (4.5)	0.007*
DM II + skin dis	1 (2.9)	0 (0.0)	0 (0.0)	0 (0.0)	1 (0.9)	0.525
Congenital syndromes	1 (2.9)	2 (5.9)	0 (0.0)	0 (0.0)	3 (2.7)	0.483
Chronic corticosteroid use	0 (0.0)	1 (2.9)	0 (0.0)	0 (0.0)	0 (0.0)	0.525

*CI* cochlear implant, *DM II* Diabetes Mellitus type II, *Skin dis.* skin disease

^a^Radiotherapy after implantation; *Represents significant difference

### Treatments and consultations per device

The total number of treatments and incidence of consultations over 5 years are presented in Table 3 and Supplement 1a, b.

**Table 3:** Incidence and costs of additional post-implantation treatment per device over 5 years

Type of treatment	pBCD	t_pas_BCD	t_act_BCD	CI
Antibiotics (cures)	5	13	2	3
	€12.5	€56.2	€5.0	€7.6
Painkillers (tramadol/oxycodone)	0	36	60	0
	€0.0	€3.6	€6.0	€0.0
TCPB ointment	91*	2	0	2
	€271.0	€6.0	€0.0	€6.0
Fucidin ointment	1	34^a^	0	2
	€3.0	€102.0	€0.0	€6.0
Healing caps	37*	0	0	0
	€1591.0	€0.0	€0.0	€0.0
Emergency room consult	1	1	1	1
	€484.0	€484.0	€484.0	€484.0
Soft tissue revision	1	0	0	0
	€484.0	€0.0	€0.0	€0.0
Revision surgery	1	0	0	0
	€2220.9	€0.0	€0.0	€0.0
Postoperative complications (surgery)	1	0	0	0
	€2220.9	€0.0	€0.0	€0.0
Ward admission	1	0	0	0
	€851.0	€0.0	€0.0	€0.0
Implant removal	1	7	0	0
	€2220.9	€15,546.3	€0.0	€0.0
Pain specialist + anesthetic injection	0	1	0	0
	€0.0	€242.0	€0.0	€0.0
External magnets	n/a	21	9	0
	€0.0	€2268.0	€288.0	€0.0
New/changed abutment	2	0	0	0
	€2412.0	€0.0	€0.0	€0.0
Surgical repositioning of implant	1	0	0	0
	€2220.9	€0.0	€0.0	€0.0

*n/a* not applicable

^a^34 times prescribed as per protocol

### System comparisons—total post-implantation costs

**Fig. 1:**
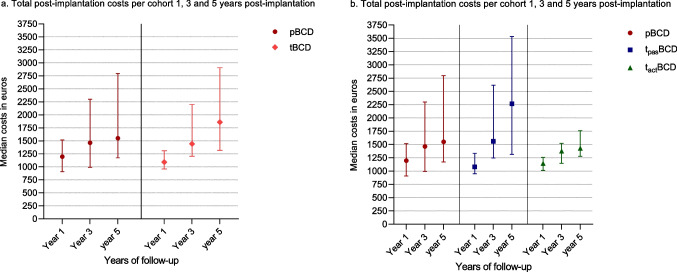
**a**, **b** Point plot of median and interquartile range of total post-implantation cumulative costs per cohort and type of device are shown at all moments of follow-up. At all moments of follow-up between the pBCD and transcutaneous BCDs, no significant differences in median total post-implantation costs were found

The median total post-implantation costs in the pBCD cohort were higher compared to the tBCD cohort after 1 (*p* = 0.735) and 3 (*p* = 0.412) years, however, lower after 5 years (*p* = 0.351) (Table 4). The pBCD cohort costs were higher after 1 year (*p* = 0.816), but lower after 3 (*p* = 0.225) and 5 years (*p* = 0.170) compared to the t_pas_BCD cohort. None of these differences were statistically significant (Fig. 1). The pBCD cohort neither showed any significant different total median post-implantation costs compared to the t_act_BCD cohort at all time points: year 1 (*p* = 0.676), 3 (*p* = 0.571) and 5 (*p* = 0.550) (Fig. 1).

**Table 4:** System comparisons—total post-implantation costs

Device	Year 1	Year 3	Year 5
pBCD	€1196.1 (908.9–1518.2)	€1464.2 (992.3–2300.2)	€1550.7 (1174.6–2797.4)
tBCD	€1089.9 (959.3–1309.5)	€1442.8 (1206.0–2200.4)	€1860.0 (1317.1–2907.7)
*t*_*pas*_*BCD*	*€1080.0 (949.9–1333.6)*	*€1558.9 (1248.0–2617.9)*	*€2266.9 (1314.1–3535.3)*
*t*_*act*_*BCD*	*€1141.7 (10,112–1258.2)*	*€1374.8 (1146.6–1522.0)*	*€1428.8 (1277.3–1760.4)*

Results presented in median (interquartile range)

### *pBCD vs t*_*pas*_*BCD*

**Fig. 2:**
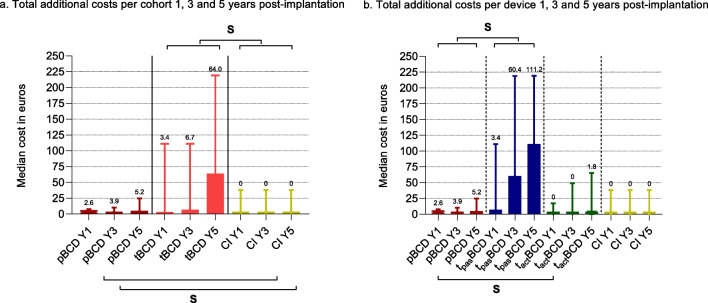
**a** Bargraph of median post-implantation additional costs per cohort. No significant differences found between pBCD and tBCD at all timepoints: year 1 (*p* = 0.191), 3 (*p* = 0.107), 5 (*p* = 0.119). Significant higher additional costs found in tBCD cohort compared to CI: year 1 (*p* = 0.016), 3 (*p* = 0.001), 5 (*p* =  < 0.001). **b** Bargraph of median post-implantation additional costs per device. Significant differences were found between the pBCD and t_pas_BCD after year 1 (*p* = 0.008), 3 (*p* = 0.007) and 5 (*p* = 0.021). Furthermore, after 1 year, a significant difference was shown between pBCD and t_act_BCD (*p* = 0.010). The CI cohort’s median was significantly lower compared to the pBCDs after 3 (*p* = 0.043) and 5 years (*p* = 0.019). Numbers represent median additional costs. IQR presented in T-plot. ‘S’ represents a significant difference

Median additional post-implantation costs between the pBCD and the t_pas_BCD cohort were significantly lower after 1 (*p* = 0.008), 3 (*p* = 0.007) and 5 years (*p* = 0.021) (Table 5; Fig. 2). Between the pBCD and the t_pas_BCD cohorts, no significant differences were found in median consultation costs after 1 (*p* = 0.548), 3 (*p* = 0.345) and 5 years (*p* = 0.239).

**Table 5:** System comparisons—additional and consultation costs separated per device

	Year 1	Year 3	Year 5
pBCD versus tBCD			
	Additional costs		
pBCD	€2.6 (2.6–7.7)	€3.9 (2.6–10.4)	€5.2 (2.6–24.8)
tBCD	€3.4 (3.4–111.2)	€6.7 (3.4–111.2)	€64.0 (3.4–219.1)
	Consultation costs		
pBCD	€1993.5 (906.4–1510.6)	€1392.4 (987.9–2069.4)	€1459.8 (1105.1–2755.9)
tBCD	€1141.7 (906.2–1292.9)	€1379.0 (1157.1–1937.7)	€1677.1 (1294.8–2509.5)
	Medical consultation costs		
pBCD	€138.5 (102.0–274.3)	€271.8 (136.3–408.6)	€271.9 (136.4–490.7)
tBCD	€336.9 (138.3–407.4)*	€543.2 (271.8–611.1)*	€543.2 (271.8–739.0)*
	Audiological consultation costs		
pBCD	€1024.8 (731.3–1319.0)	€1155.6 (819.3–1717.4)	€1331.8 (953.1–2012.0)
tBCD	€770.4 (548.6–940.6)*	€1057.5 (770.4–1293.0)	€1162.4 (835.7–1762.6)
pBCD versus t_pas_BCD			
	Additional costs		
pBCD	€2.6 (2.6–7.7)	€3.9 (2.6–10.4)	€5.2 (2.6–24.8)
tBCD	€3.4 (3.4–111.2)*	€60.4 (3.4–219.1)*	€111.2 (3.4–219.3)*
	Consultation costs		
pBCD	€1993.5 (906.4–1510.6)	€1392.4 (987.9–2069.4)	€1459.8 (1105.1–2755.9)
tBCD	€1064.3 (876.8–1302.2)	€1464.1 (1233.3–2234.5)	€1892.4 (1294.3–2766.0)
	Medical consultation costs		
pBCD	€138.5 (102.0–274.3)	€271.8 (136.3–408.6)	€271.8 (136.4–490.7)
tBCD	€404.8 (271.6–473.3)*	€543.2 (374.1–740.3)*	€544.5 (406.8–745.0)*
	Audiological consultation costs		
pBCD	€1024.8 (731.3–1319.0)	€1155.6 (819.3–1717.4)	€1331.8 (953.1–2012.0)
tBCD	€770.4 (548.6–904.6)*	€1024.8 (714.9–1668.5)	€1240.7 (770.4–2095.8)
pBCD versus t_act_BCD			
	Additional costs		
pBCD	€2.6 (2.6–7.7)	€3.9 (2.6–10.4)	€5.2 (2.6–24.8)
T_act_BCD	€0.0 (0.0–17.3)*	€0.0 (0.0–49.3)	€1.8 (0.0–65.3)
	Consultation costs		
pBCD	€1193.5 (906.4–1510.6)	€1392.4 (987.9–2069.4)	€1459.8 (1105.1–2755.9)
T_act_BCD	€1149.1 (906.2–1242.2)	€1342.8 (1093.2–1488.8)	€1342.8 (1223.9–1645.7)
	Medical consultation costs		
pBCD	€138.5 (102.0–274.3)	€271.8 (136.3–408.6)	€271.9 (136.4–490.7)
T_act_BCD	€135.8 (135.8–306.7)	€271.6 (135.8–374.6)	€274.3 (135.8–405.8)
	Audiological consultation costs		
pBCD	€1024.8 (731.3–1319.0)	€1155.6 (819.3–1717.4)	€1331.8 (953.1–2012.0)
T_act_BCD	€875.4 (875.4–973.3)	€1071.2 (875.4–1234.8)	€1136.4 (940.6–1234.8)
pBCD versus CI			
	Additional costs		
pBCD	€2.6 (2.6–7.7)	€3.9 (2.6–10.4)	€5.2 (2.6–24.8)
CI	€0.0 (0.0–38.1)	€0.0 (0.0–38.1)*	€0.0 (0.0–38.1)*

Results presented in median (interquartile range)

*Represents significant difference

**Fig. 3:**
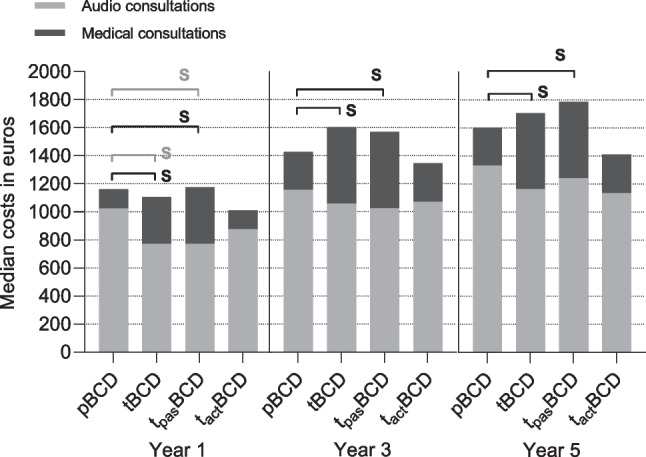
Bargraph of median post-implantation consultation costs divided by consultation type. Medical consultations were significantly lower in the pBCD cohort compared to the tBCD and t_pas_BCD cohort at all timepoints, respectively Y1: *p* = 0.010, p = 0.002; Y3: *p* = 0.005, p < 0.001; Y5: *p* = 0.008, *p* = 0.001); no differences were found between the pBCD and t_act_BCD cohort. After 1 year, significant differences were found in audiological consults between the pBCD, tBCD (*p* = 0.033) and t_pas_BCD cohorts (*p* = 0.020). After 1, 3 and 5 years, no significant differences were also found in audiological consultations between the pBCDs and t_act_BCDs (*p* = 0.570; *p* = 0.420; *p* = 0.310). Significant differences in medical consultations are represented by black ‘S’; significant differences in audiological differences are represented by grey ‘S’

Dividing the total consultation costs in medical and audiological consults, the medical consultations for the pBCD cohort compared to the t_pas_BCD cohort were significantly lower at all time points (Y1: *p* = 0.002; Y3: *p* < 0.001; Y5: *p* = 0.001) (Table 5; Fig. 3). Conversely, the audiological consultations were significantly higher for the pBCDs compared to the t_pas_BCDs after 1 year (*p* = 0.020) and broadly similar after 3 (*p* = 0.314) and 5 years (*p* = 0.650).

### *pBCD vs t*_*act*_*BCD*

Median additional costs were higher in the pBCD cohort compared to the t_act_BCD after 1 (*p* = 0.010), 3 (*p* = 0.066) and 5 years (*p* = 0.295), with the only significant difference being after year 1 (Table 5; Fig. 2). After 1 (*p* = 0.591), 3 (*p* = 0.571) and 5 years (*p* = 0.676), the median consultation costs of the pBCD cohort were not significantly higher.

The medical consultation costs compared between the pBCD and t_act_BCD cohorts showed broadly similar costs after 1 (*p* = 0.964), 3 (*p* = 0.869) and 5 years (*p* = 0.846) (Table 5; Fig. 3). The audiological consultations were not significantly higher for the pBCDs compared to the t_act_BCDs after all time points (Y1: *p* = 0.570; Y3: *p* = 0.420; Y5: *p* = 0.310).

### pBCD vs CI

The pBCD cohorts’ median additional post-implantation costs were non-significantly higher after 1 (*p* = 0.057) year, but significantly higher after 3 (*p* = 0.043) and 5 (*p* = 0.019) years compared to the CI cohort (Table 5; Fig. 2).

## Discussion

### Key findings and interpretation

With increasing availability and improvement of transcutaneous solutions, it is crucial to evaluate and compare costs of bone conduction devices (BCDs), especially since transcutaneous linked devices are more expensive but might become cost-beneficial over time [11, 12]. This study revealed that in the Radboudumc the total post-implantation cost of percutaneous BCDs (pBCD, i.e. BIA300®) was statistically not significantly different from transcutaneous BCDs (tBCD). Additionally, cost in the pBCD cohort did not differ significantly from passive tBCDs (t_pas_BCD, i.e. Baha® Attract), and active transcutaneous BCDs (t_act_BCD, i.e. Osia® I). Audiological consultations largely influenced the post-implantation cost (Fig. 3). The additional costs were minimal for all devices following little complications, although the t_pas_BCD showed more costs in comparison.

### Additional costs

The additional post-implantation costs of the pBCD and tBCD did not differ significantly at any moment of follow-up, even though the median costs in the transcutaneous cohort were slightly higher. Reason for this result were the relatively cheaper interventions and treatments admitted in the percutaneous cohort. Furthermore, the t_pas_BCDs were responsible for a large part of the cost in the tBCD aggregated cohort.

In the t_pas_BCDs the additional costs were statistically significant higher at all follow-up moments compared to the pBCDs (Fig. 2). These higher costs may be explained by a moderate correlation between the number of t_pas_BCD explantations (6; 17.6%) and the costs associated with complications and interventions over 5 years (*r* = 0.599, *p* < *0.001*). Three of these explantations were conversions to a percutaneous device. Interestingly, the additional cost in the pBCD and t_pas_BCD cohorts were relatively low compared to the consultations, respectively, adding up to €5.2 and €111.2 over 5 years, having a lesser impact on the total cost compared to the consultations (Figs. 2 and 3).

The t_act_BCD cohort did have statistically significant lower additional costs compared to the pBCDs after 1 year, meaning less post-implantation treatment was needed. This corresponds with previous studies by Gawecki et al. and Lau et al. stating few complications during the first year after implantation with an t_act_BCD [13, 14]. After 3 and 5 years, the additional cost was broadly similar meaning few treatments in both cohorts. However, note that the heterogeneity was quite large in the t_act_BCD cohort.

During 5 years, the cochlear implant (CI) users needed very little medical treatment. The most common complications reported in CI users are pressure-related erythema or skin defects (due to magnet) and skin flap necrosis, which are rarely reported [15] and were not observed in this current study. This underlines the transcutaneous’ link low vulnerability, connecting to the internal implant receiver that is similar to the t_act_BCD.

### Complications

The percentage of adverse skin reactions -using the Holgers’ score (grade 2–4) since the IPS-scale[16] was not already introduced—calculated over all 164 observations in the pBCDs was 11.0% compared to 6.5% of a random sample of 34 subjects taken from the cohort from Dun et al. (surgery age 18 + ; mean follow-up 4.6 years) [5]. The higher percentage of 11.0% overall observations can be explained by two patients in whom eight of the 18 adverse skin reactions were observed (44.4%). The surgical revision rate was comparable to the cohort from Dun et al. (*n* = 34), respectively 17.6% versus 20.6% [5]. Reasons for revision surgery were postoperative complications (*n* = 1), abutment replacements (*n* = 2) and removal (*n* = 1), soft tissue revision (*n* = 1) and reimplantation due to a complication (*n* = 1), whereas in the cohort from Dun et al., the reasons were skin reduction (*n* = 4), skin revision (*n* = 2) and abutment removal (*n* = 1). No implants were lost in this study’s cohort. This is a complication—for reference purposes—occurring in approximately 0.6–17.4% of pBCD implants [6, 17–20].

### Comparison with other studies

The pBCD (Baha® DermaLock) cohort in the study of Godbehere et al. showed a cost average of £903 (i.e. €1087, not taking inflation since 2014 into account) per subject over 6 months, not including the initial purchase of the device (£5103.6) and surgery (£1516.6) [12]. This is comparable with the median post-implantation total of €1196 for the pBCDs after one year in the current study. Within their t_pas_BCD cohort (Baha® Attract), the cost total was £502 (i.e. €604) after 6 months, excluding purchase (£5225.4) and surgery (£1516.59). In this current study, a cost total of €1080 was observed after 1 year. Reasons for this difference between studies are a 6-month longer follow-up, a more detailed reported number of clinical consultations and two patients having revision surgery during the first year in this current study.

In the study of Godbehere et al., the pBCDs were €483 more expensive than t_pas_BCDs, whereas in the current study, this difference was €116. Their pBCD cohort needed more out-patient consultations compared to the t_pas_BCDs. Considering a 6-month follow-up, Godbehere et al. reported an adverse soft tissue reactions rate (i.e. Holgers >  = 2) of 32% per patient, which is comparable to this study (26.5%) and other studies: 20–58.8% Den Besten et al. [21], 18.8–25% Kara et al. [22]. As opposed to the current study, their study mentioned a lower medical and audiological consultation rate for the t_pas_BCD cohort compared to the pBCD cohort, arguably due to fewer skin complications. The 6-month follow-up is a limiting factor since more implant-related issues might be expected afterwards, however, there is little literature available concerning adult t_pas_BCD patients followed up for multiple years [23].

In a more recent study, Amin et al. compared t_act_BCDs (BoneBridge 601, MED-EL, Innsbruck, Austria) with pBCDs and concluded that the t_act_BCD became cost-beneficial 5 years after implantation. After 5 years, the mean total cost in the pBCD cohort, subtracted by the initial implant purchase (£1040), sound processor (£2356) and surgical costs (£401) was £8778 (i.e. €10,570), whereas the t_act_BCD cost was £3493 (i.e. €4206). This results in a difference of €6364, with the pBCD being clinically much more expensive than the transcutaneous counterpart. In the current study, this difference after 5 years was only €122. Reasons for the large difference in the study of Amin et al. could be significantly more wound care appointments and requirements, a higher surgery revision rate and a comparable amount of sound processor upgrades while the pBCD sound processor was more expensive. Reasons for the non-significant difference in this current study were the low revision surgery rate, 0% of subjects needing revision surgery more than once and only two abutment changes during 5 years of follow-up. Additionally, sound processor upgrades were dismissed. Conversely, in the study of Amin et al., 36% of subjects needed revision surgery more than once and seven abutment changes were performed. Audiological consultations were significantly higher after 1 and 3 years, due to more repairs and programming although these exact numbers were not presented. The current study presents the opposite with the pBCDs needing more audiological adjustments, whereas the t_act_BCD is most expensive during the first year and relatively problem free and consistent afterwards. Interestingly, the results of Amin et al. show no significant differences after 5 years, including implant/processor purchase and surgery, equivalent to the current study.

### Strengths and limitations

Even though not being the first study to perform a clinical cost analysis, it gives the nearest possible insight into the total cost differences between percutaneous and transcutaneous BCDs, related to post-operative care, and the rehabilitation process over a long (5-year) follow-up period. The comparison between the CI reference cohort and pBCD cohort emphasizes the advantageous effect of an active transcutaneous link design. Furthermore, by evaluating medical and audiological consultations separately, it was shown that the audiological follow-up has a major influence on the total cost of both types of BCDs. Two of the passive transcutaneous devices that were explanted during follow-up were replaced by percutaneous devices. The costs related to their percutaneous device were included in further follow-up in the t_pas_BCD cohort.

A note must be taken when interpreting and comparing the results of the pBCD (*n* = 34) and t_act_BCD (*n* = 9) cohort due to the skewness in sample size. Reason for having nine t_act_BCD patients is that these are the only first generation Osia patients in our centre having passed their 5-year follow-up. For this reason, comparisons between pBCDs and the tBCDs as a whole were reported as well. Additionally, all patients were consecutively chosen instead of random, increasing risk of selection bias. Moreover, the number of upgraded sound processors was significantly lower in the pBCD cohort compared to the transcutaneous device cohorts and not included in analysis; respectively, 7 pBCDs (21%), 14 t_pas_BCDs (41%) and 9 t_act_BCDs (100%). Even though patients in the Radboudumc are allowed to upgrade their sound processor approximately five years after implantation, many do not and wait another 1 or 2 years. If hypothetically all sound processors (average costing €5000) in this cohort would be replaced after 6 or 7 years, the yearly cost reduction per patient would be €167 and €286 after 6 and 7 years, respectively. This means that the effect of delaying sound processor replacement is potentially more influential than the actual differences in medical and audiological consultations between pBCDs and tBCDs.

A limitation is that the t_act_BCD and most t_pas_BCD were newly implemented during a trial. Therefore, audiological consultations during fitting were inventoried by current protocol. It is feasible that after years of experience audiologists might change their routine performing fewer tests and encounter fewer problems.

The data used for analysis were gathered retrospectively. Due to this reason, the investigators were reliable on the record-keeping by clinicians. In addition, the cohorts’ subjects were not randomly chosen but picked consecutively.

Finally, the cohorts solely existed out of adults, whereas children tend to show more adverse skin reactions and implant losses. Therefore, the additional treatment costs and medical consultation costs, especially in the pBCD cohort, can be underestimated.

### Clinical applicability

At the Radboudumc, percutaneous solutions are still the gold standard in patients with an indication for a bone conduction device, both from a medical as well as an audiological perspective. However, tBCDs, specifically t_act_BCDs, are indicated more frequently because of existing skin issues and the preference of the candidate. Moreover, it can be hypothesized that with increasing clinical experience the cost of t_act_BCDs will become less after 5 years, making them less expensive compared to pBCDs.

Patients fitted with a pBCD, t_pas_BCD, t_act_BCD and CI required limited additional care after implantation, although higher costs were seen in t_pas_BCDs. Due to the higher cost combined with reported limited output [24], t_pas_BCDs appear less beneficial for patients, usually leading to the decision for a t_act_BCD in our centre.

Considering the results, it is clear that medical and audiological post-implantation treatments and consultations are broadly similar between pBCDs and t_act_BCDs after 5 years, meaning initial purchase and surgery (still) have a large impact on total cost. This also indicates that pBCD performs well regarding soft tissue reactions and implant longevity [25].

From a caretaker’s perspective, when consulting a patient there are multiple considerations taken into account such as differences in output, soft tissue reactions, MRI compatibility and related comorbidities, incision types and scarring and anaesthesia. All these factors should outway the results of this study in decision making. Finally, this study did not find hard evidence preferencing pBCDs or t_act_BCDs in terms of costs.

## Conclusion

Total post-implantation costs were not significantly different between the percutaneous and transcutaneous (either active or passive) bone conduction devices. Passive transcutaneous bone conduction devices showed significantly higher complication costs after implantation due to more explantations.

### Supplementary Information

Below is the link to the electronic supplementary material.

Supplementary file1 (DOCX 19 KB)

## Data Availability

The authors confirm that the data supporting the findings in this study are available within the article and supplementary material. Raw data used to support the findings of this study are available from the corresponding author, upon reasonable request.
