# Supplementary material for: Post-implantation clinical cost analysis between transcutaneous and percutaneous bone conduction devices
Source: Eur Arch Otorhinolaryngol. 2023 Jul 8;281(1):117–27. doi: 10.1007/s00405-023-08099-2 (PMC10764476; doi:10.1007/s00405-023-08099-2)
Supplement: Supplementary file 1 — Supplementary file1 (DOCX 19 KB) [file 405_2023_8099_MOESM1_ESM.docx]

| Supplement 1a: Incidence of post-implantation consultations per device over five years | | | | | | | | | |
| --- | --- | --- | --- | --- | --- | --- | --- | --- | --- |
| **pBCD (n=34)** | | **tBCD (n=43)** | |  | |  | | **CI (n=34)** | |
|  | |  | | ***t_pas_BCD (n=34)*** | | ***t_act_BCD (n=9)*** | |  | |
| **Number** | **Mean** | **Number** | **Mean** | ***Number*** | ***Mean*** | ***Number*** | ***Mean*** | **Number** | **Mean** |
| TN: 19 TD: 4 TA: 46 PN: 128  PD: 79  PAC: 12 PAS: 135  PAE: 63  TOT: 486 | (0.6)  (0.1)  (1.4)  (3.8)  (2.3)  (0.4)  (4.0)  (1.9)  (14.3) | TN: 17 TD: 44 TA: 45 PN: 31  PD: 173  PAC: 4 PAS: 119  PAE: 93  TOT: 526 | (0.4)  (1.0)  (1.0)  (0.7)  (4.0)  (0.1)  (2.8)  (2.2)  (12.2) | *TN: 13 TD: 38 TA: 26 PN: 30*  *PD: 157*  *PAC: 2 PAS: 107*  *PAE: 74*  *TOT: 447* | *(0.4)*  *(1.1)*  *(0.8)*  *(0.9)*  *(4.6)*  *(0.1)*  *(3.2)*  *(2.2)*  *(13.1)* | *TN: 4*  *TD: 6*  *TA: 19*  *PN: 1*  *PD: 16*  *PAC: 2*  *PAS: 12*  *PAE: 19*  *TOT: 79* | *(0.4)*  *(0.7)*  *(2.1)*  *(0.1)*  *(1.8)*  *(0.2)*  *(1.3)*  *(2.1)*  *(8.8)* | TN: 3  TD: 1  PN: 29  PD: 31  TOT: 64 | (0.1)  (0.03)  (0.9)  (0.9)  (1.9) |

| Supplement 1b: comparisons of post-implantation consultations per device over five years | | | | | | | | | |
| --- | --- | --- | --- | --- | --- | --- | --- | --- | --- |
| **pBCD vs tBCD** | | **pBCD vs t_pas_BCD** | | **pBCD vs t_act_BCD** | | **t_pas_BCD vs t_act_BCD** | | **pBCD vs CI** | |
| **Type** | ***p*-values** | **Type** | ***p*-values** | **Type** | ***p*-values** | **Type** | ***p*-values** | **Type** | ***p*-values** |
| TN TD* TA PN*  PD*  PAC PAS*  PAE  TOT | 0.176  <.001*  0.081  <.001*  0.005*  0.073  0.018*  0.069  0.178 | TN TD* TA* PN*  PD*  PAC* PAS  PAE  TOT | 0.141  <.001*  0.018*  <.001*  <.001*  0.036*  0.189  0.150  0.439 | TN TD* TA* PN*  PD  PAC PAS  PAE  TOT | 0.731  0.017*  0.547  <.001*  0.819  0.840  <.001*  0.068*  0.042* | TN TD TA* PN*  PD*  PAC PAS  PAE  TOT | 0.557  0.554  0.057  0.013*  0.002*  0.138  0.003*  0.521  0.085 | TN* TD  PN*  PD*  TOT* | <.001*  0.166  <.001*  <.001*  <.001* |
| Abbreviations: TN, telephonic nurse consultation; TD, telephonic doctor consultation; TA, telephonic audiological consultation; PAC, physic audiological consultation; PAS, physic audiological simple consultation; PAE, physic audiological extended consultation; PN, physic nurse consultation; PD, physic doctor consultation; TOT, total hospital contacts.  Asterisk (*) represents a statistically significant difference  *P*-values calculated with Mann-Whitney U test. | | | | | | | | | |
